# Discontinuation of simvastatin leads to a rebound phenomenon and results in immediate peri‐implant bone loss

**DOI:** 10.1002/cre2.23

**Published:** 2016-03-18

**Authors:** Xianqi Li, Feng Wu, Yiming Zhang, Jing Yang, Atsushi Shinohara, Hideaki Kagami

**Affiliations:** ^1^ Department of Hard Tissue Research, Institute for Oral Science Matsumoto Dental University Shiojiri Japan; ^2^ School of Dentistry Shanxi Medical University Taiyuan China; ^3^ Center of Health‐Care in Stomatology Tenth People's Hospital of Tongji University Shanghai China; ^4^ Department of Oral Health Promotion, Institute for Oral Science Matsumoto Dental University Shiojiri Japan; ^5^ Department of Oral and Maxillofacial Surgery, School of Dentistry Matsumoto Dental University Shiojiri Japan

**Keywords:** Bone loss, implant, micro‐CT, osteogenesis, rebound phenomenon, simvastatin

## Abstract

Although administration of simvastatin has been reported to promote bone formation, the effect of short‐term simvastatin administration is not well known. Following implant installation, 10‐week‐old male Wistar rats (*n* = 24) were divided into two groups randomly. The experimental group received 10 mg/kg of simvastatin daily for seven days. Then simvastatin administration was discontinued, and the animals were observed up to 28 days. Animals in the control group underwent the same procedure but received saline instead of simvastatin. All animals were analyzed by micro‐computed tomography. Samples at days 14 and 21 were subjected to histological analyses. After seven days of simvastatin administration, more new bone formation around the implant was observed in the simvastatin group compared with the control group. Seven days after simvastatin discontinuation, however, the amount of peri‐implant trabecular bone began to decrease. Results from morphometric analysis also showed a reduction in new bone area after day 7, which was lowest at day 14. These results were confirmed by histological analyses. In contrast, both the peri‐implant trabecular bone and new bone area were maintained in the control group. Short‐term administration of simvastatin may affect implant stability owing to a rebound phenomenon and an immediate loss of peri‐implant bone.

## Clinical Relevance

Scientific rationale for the study: Administration of simvastatin has been reported to promote bone formation, as well as bone‐to‐implant contact around implants. However, no study investigated the impact of short‐term simvastatin administration and subsequent discontinuation on peri‐implant bone formation.
Principal findings: Discontinuation of simvastatin after short‐term administration causes immediate bone loss and may affect implant stability.
Practical implications: A favorable effect was not demonstrated in dental implant therapy by short‐term administration of simvastatin.

## Introduction

Much research has focused on improving early osteogenesis and shortening the period of bone healing around dental implants. To date, several modified methods involving implant materials with improved topographical and chemical properties have been shown to shorten the period of bone healing (Botticelli et al., 2006; Butz et al., 2006; Avila et al., 2009; Fajardo et al., 2010; Albertini et al., 2015). A strategy that also improves osteogenesis around implants is highly desirable.

Simvastatin is a statin that inhibits 3‐hydroxy‐3‐methylglutaryl coenzyme A reductase. Simvastatin has been widely used for the treatment of dyslipidemia (Nozue and Michishita, 2015) and in the prevention of cardiovascular disease (Soran et al., 2015). Recent studies have shown that simvastatin may also promote osteogenesis by inducing bone morphogenetic protein 2 (BMP‐2) in osteoblasts (Mundy et al., 1999; Ho et al., 2009). Accordingly, systemic administration of simvastatin has been reported to increase bone mineral density in osteoporotic patients, thus reducing the risk of fracture (Horiuchi and Maeda, 2006; Helin‐Salmivaara et al., 2012; Thabit et al., 2014). The ability to promote osteogenesis is a desirable property of dental implant treatments, as it can enhance both new bone formation and the bone‐to‐implant contact (BIC) when applied locally (Ma et al., 2008; Fang et al., 2015) and systematically (Du et al., 2009; Ayukawa et al., 2010).

Simvastatin has been associated with various side effects, such as dual effect of periodontium (Saxlin et al., 2009), muscular adverse effects (Golomb and Evans, 2008), liver damage (Covelli et al., 2015), and exaggeration of diabetes (Enas et al., 2013). Owing to these long‐term side effects, effective short‐term simvastatin administration could be beneficial. In addition, a number of the aforementioned studies utilized two‐dimensional histological analyses, which may be unsuitable for the analysis of the three‐dimensional bone structure around implants.

In this study, we focused on the effect of short‐term systemic administration of simvastatin on peri‐implant bone formation, as well as the effect of simvastatin discontinuation on bone formation. An animal model was used in combination with micro‐computed tomography (micro‐CT) and specialized software for three‐dimensional histological analysis.

## Materials and Methods

### Animals

The animals were treated according to the guidelines of animal care. This study was carried out under the approval of the Animal Management Committee of Matsumoto Dental University (approval number 143, 211–12).

Male Wistar rats that are 10 weeks old (*n* = 24, weighing 220–250 g at the beginning of the experiments) were obtained from an animal resource center (Japan SLC, Inc., Shizuoka, Japan). All rats were housed in a specific‐pathogen‐free, temperature‐controlled room under a 12‐h alternating light–dark cycle and given free access to food and water.

### Implants and implantation

**Figure 1:**
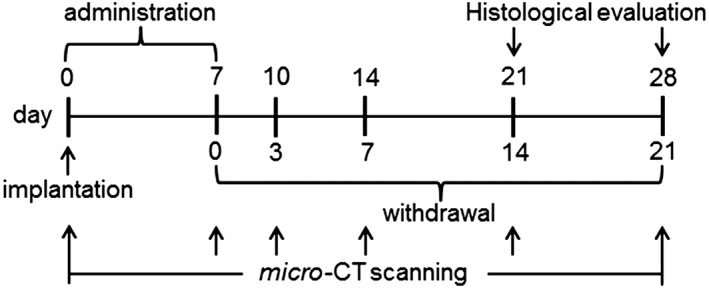
Experimental schedule for the present study.

**Figure 2:**
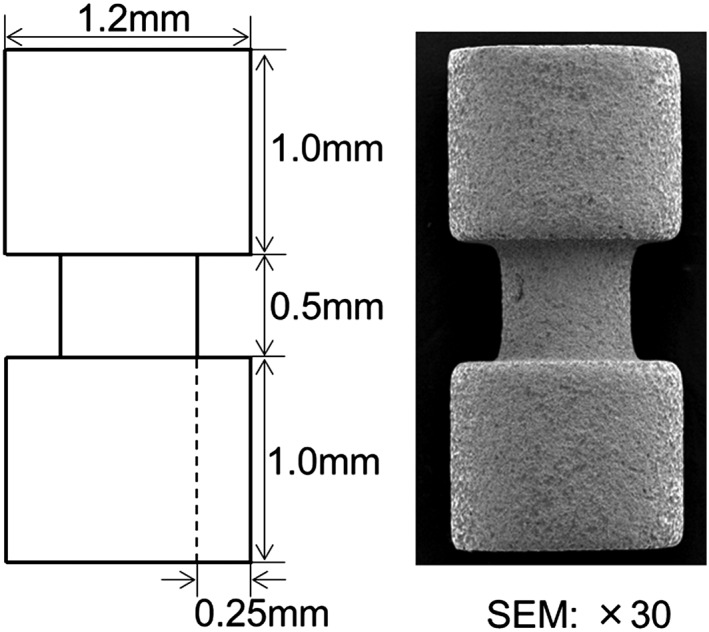
Design of the experimental implant.

The experimental schedule is summarized in Figure 1. Commercially available implants (measuring 2.5 mm in length and 1.2 mm in diameter) made from pure titanium, which had been sand blasted and acid etched, were used in this study (GC Co., Tokyo, Japan) (Fig. 2). Implant installation was performed under general anesthesia with intraperitoneal administration of pentobarbital sodium (65 mg/kg, Somnopentyl™; Kyoritsu Seiyaku Corp., Tsukuba, Japan). After incision of the skin and elevation of the periosteal flap, an implant socket was generated 5 mm inferiorly from the apical region of the knee joint, at the right (and left) hind tibia. A socket measuring 1.2 mm in diameter was prepared by drilling (drill rotary speed: 800 rpm) from the lateral to the medial side of the tibia. One implant was placed in the prepared implant socket in each leg. Following implantation, the periosteal flaps were sutured into appropriate positions.

### Simvastatin administration

All animals were randomly assigned to one of two groups. Immediately after implant installation, animals in the experimental group received daily intraperitoneal injections of 10 mg/kg of simvastatin (TCI, Tokyo, Japan) for seven days. This dosage is in accordance with previous studies (Mundy et al., 1999; Ayukawa et al., 2010). Animals in the control group received the same amount of saline, instead of simvastatin, at the same time points as the animals in the experimental group.

### Micro‐CT analysis

Under general anesthesia as described earlier, the micro‐CT (Rigaku Co., Tokyo, Japan) analysis was carried out at 0, 7, 14, 21, and 28 days after implantation.

#### Imaging system

The micro‐CT system was composed of a microfocus X‐ray tube with a focal point of 7 µm (L9181S; Hamamatsu Photonics, Hamamatsu, Japan). The X‐ray sensor contained a 4‐in. image intensifier. The exposure parameters were 100 kV and 160 μA, and the scan time was 2 min. Image reconstruction was carried out on a personal computer using the specially designed i‐view software 1.68 (J. Morita, Kyoto, Japan).

#### Morphometric analysis

Bone volume (BV) around the implant was measured from voxel images using the sub‐XYZ software (Arai Y., Matsumoto Dental University, Japan), a specially designed BV‐measuring software, as described by Kochi et al. (Kochi et al., 2009). Briefly, gray values and the number of voxels displaying a particular gray value were calculated in regions of interest (ROI) using the BV‐measuring software, and a histogram of the X‐ray absorption rate was calculated, which showed peaks for the hard and soft tissues in the field of view of the CT imaging area. The threshold was then set at the value for the trough between these peaks, and the number of voxels in which the X‐ray absorption rate exceeded the threshold in a CT image was considered as a measure of newly mineralized tissue (new bone). Bone tissue in the ROI was analyzed under the same conditions at each time point. The increase in bone area (new bone area) was calculated by subtracting the bone area on day 0 from that on each subsequent day. Any increase in BV was considered to be due to new bone formation.

### Histology

Rats were euthanized by administration of an overdose of anesthesia at days 14 and 21, following discontinuation of simvastatin. The tibia was excised and immediately fixed in 10% neutral buffered formalin solution, dehydrated in increasing gradients of alcohol, and embedded in a methacrylate‐based resin (Technovit 9100; Heraeus Kulzer GmbH, Wehrheim, Germany) according to the manufacturer's instructions. Sections (~200 µm thickness) were cut, aiming the center of the implant along its long axis. The thickness of each section was then reduced to <50 µm by means of a series of SiC abrasive papers under water irrigation (Donath and Breuner, 1982). The sections were stained with toluidine blue and subjected to optical microscopy for histological evaluation. BIC was measured manually from photomicrographs using the imagej software (NIH, Bethesda, MD).

### Statistical analysis

Bone volume in the experimental group was compared with that in the control group by independent‐sample tests. All analyses were performed using spss 17.0 (IBM, Chicago, IL). *P*‐values of <0.05 were considered to be statistically significant.

## Results

Healing progressed uneventfully in all animals, and no complications occurred. Moreover, no sign of postoperative infection was observed in any of the animals.

### CT images

**Figure 3:**
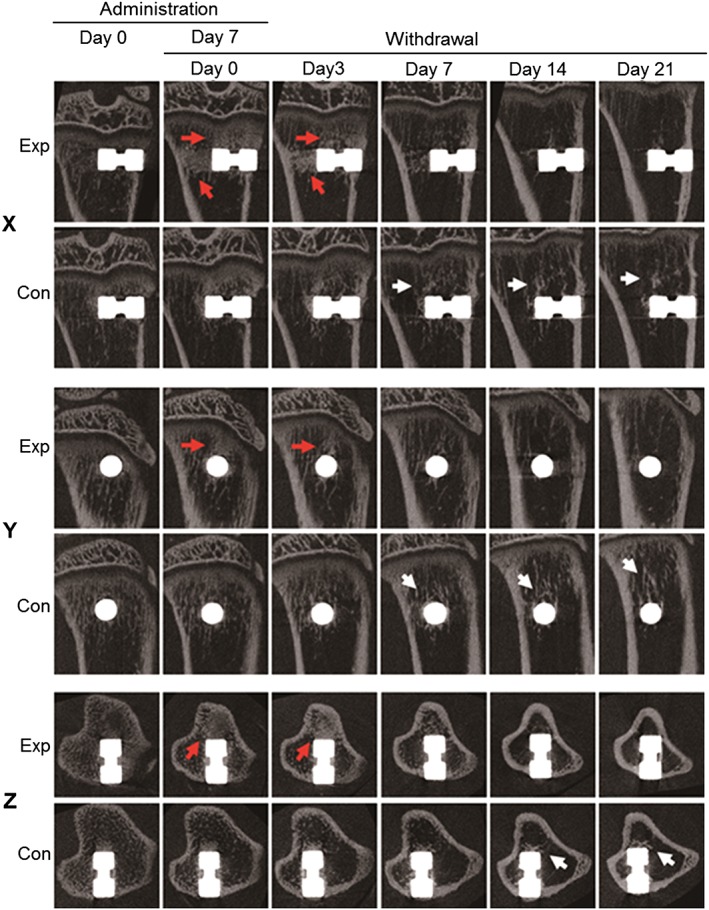
An X–Y–Z image produced using micro‐CT scans obtained before and after simvastatin discontinuation. The number of bone trabeculae around the implant clearly increased (red arrows) on days 0 and 3 in the experimental (Exp) group, compared with the control (Con) group. By day 14 after discontinuation of simvastatin, the Exp group showed a rapid decrease in the amount of trabecular bone, with the density of trabecular bone gradually worsening in most cases, whereas no marked decrease of trabecular bone was observed in the Con group (white arrows).

In the experimental group, micro‐CT images taken on days 0 and 3 following simvastatin discontinuation showed an increase in the amount of trabecular bone around the implant, with a rapid decrease observed from day 7 (Fig. 3). On the other hand, the amount of trabecular bone around the implant was maintained in the control group.

### Morphometric analysis

**Figure 4:**
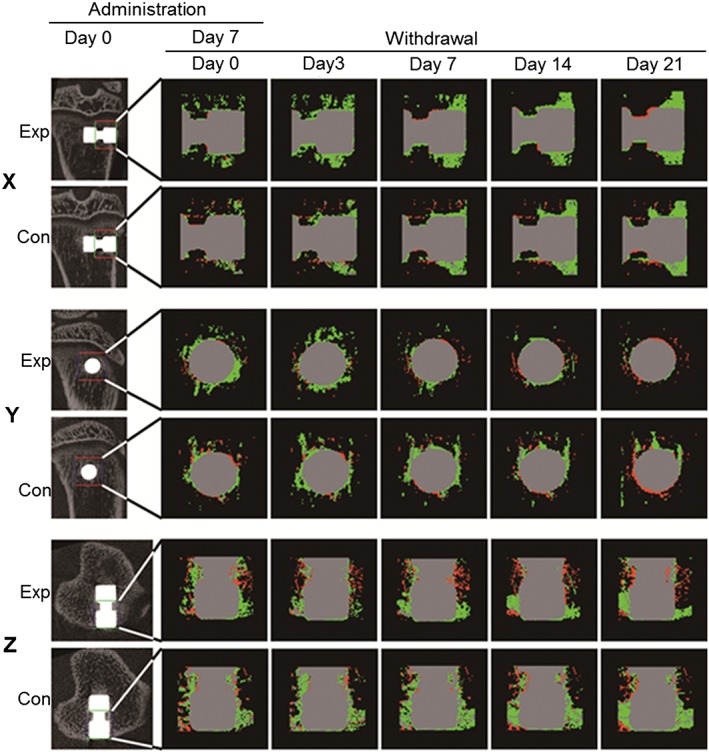
An X–Y–Z image processed for histomorphometric analysis using sub‐XYZ software. Bone volume was measured on the first postoperative day in the regions of interest (ROI) and every day thereafter under the same conditions. More newly mineralized tissue (green) was produced around the implant on days 0 and 3 in the experimental (Exp) group following discontinuation of simvastatin, compared with that in the control (Con) group, whereas on days 7, 14, and 21 following discontinuation of simvastatin, more newly mineralized tissue was produced in the Con group. ROI: 35 × 30 × 35. Green, newly mineralized tissue; red, absorbed bone; gray, no change.

Micro‐CT images were assessed by producing subtraction images to determine the level of new bone formation around the implant. The morphometric images showed that new bone area (green) around cortical bone gradually increased in both the experimental and control groups from day 0 to day 21 following implantation (as seen in the X and Z axis panels) (Fig. 4). In the experimental group, new bone area around the implant in the cancellous bone was reduced after seven days, which was more evident in the Y axis panels. A reduction in green area was observed after day 7 in the experimental group, while the green area was maintained in the control group. The absorbing area (red) in cancellous bone increased in the experimental group after day 7, with the difference being more evident in the Z axis panels.

**Figure 5:**
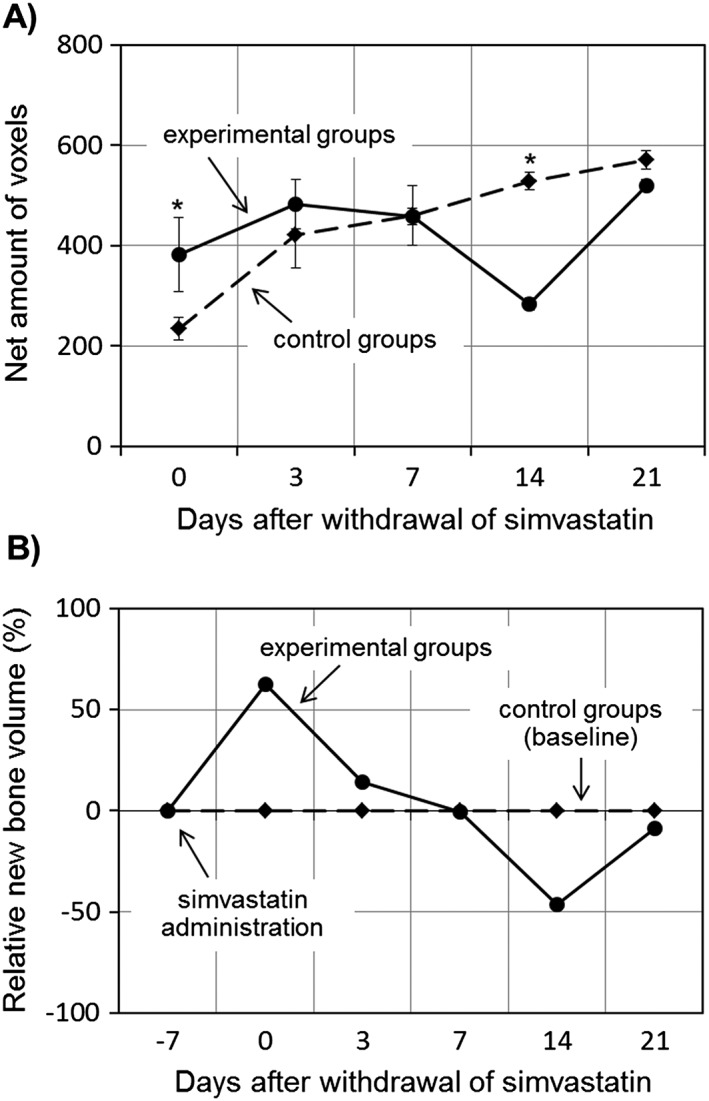
Relative changes in the amount of regenerated mineralized tissue around the implant. Following discontinuation of simvastatin, new bone volume in the experimental group increased at first, before peaking on day 3. At this point, new bone volume in the experimental group was greater than that of the control group. On day 7, new bone volume in the experimental group began to decrease, reaching its lowest value on day 14, at which it was significantly lower than that of the control group. On day 21, new bone volume in the experimental group began to increase again, reaching a similar level to that in the control group. (A) Number of voxels; (B) amount of mineralized tissue relative to the control at the same time point. *P < 0.05.

The net amount of voxels in the ROI, which represents the net amount of new bone formation around the implant, was calculated (Fig. 5A). Initially (day 0), the net amount of voxels was higher in the experimental group than in the control group (*P* < 0.05). The net amount of voxels gradually increased throughout the observation period in the control group but decreased at day 7 in the experimental group and was lowest at day 14, with the difference between days 7 and 14 being significant (*P* < 0.05). The relative new BV was calculated to visualize the difference between the experimental and control groups (Fig. 5B). A significant difference between the experimental and control groups was observed at day 3 (the value in the experimental group was higher than that in the control group) and day 14 (the value in the experimental group was lower than that in the control group) (*P* < 0.05).

### Histological examination

**Figure 6:**
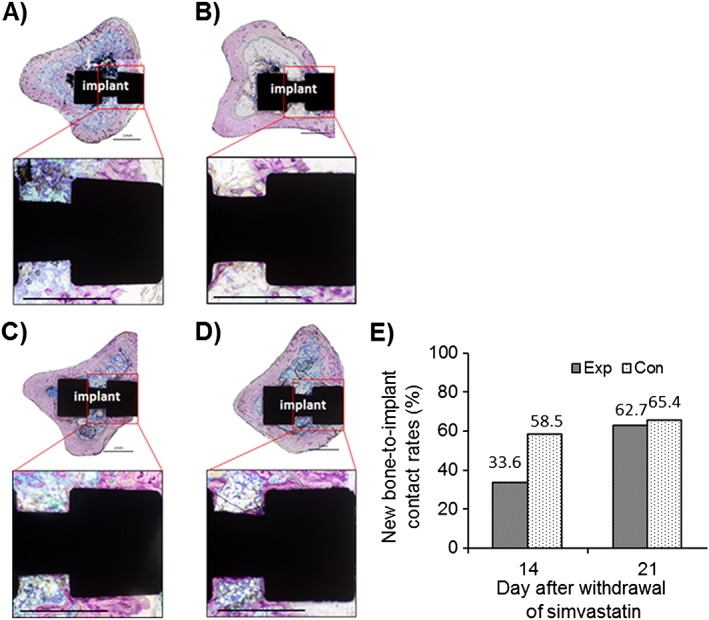
Histological observation of new bone formation around the implant on days 14 (A, B) and 21 (C, D) following discontinuation of simvastatin. New bone formation in the experimental group (A, C) was clearly lower than that in the control group (B, D). Toluidine blue staining; new bone is visualized in dark red; scale bar = 1 mm. Bone‐to‐implant contact was also measured (E) and was found to be lower in the experimental group at day 14.

Histological evaluation confirmed the results from morphometric analyses and showed less bone adjacent to the implant in the experimental groups (Fig. 6A, B), compared with that in the control groups, especially at the concave region of the implant, which faced the cancellous bone (Fig. 6C, D).

The BIC was calculated from the histological sections. Results showed less BIC in the experimental group at day 14 (Fig. 6E).

## Discussion

Recently, several studies have reported that systemic administration of simvastatin can enhance new bone formation, as well as the BIC around implants (Ma et al., 2008; Du et al., 2009; Ayukawa et al., 2010; Fang et al., 2015). Statins are known to upregulate BMP‐2 expression (Mundy et al., 1999) and promote osteoblast differentiation and mineralization (Maeda et al., 2004). In addition, it has been reported that statins also inhibit the formation and activity of osteoclasts (Hughes et al., 2007; Jeon et al., 2008; Woo et al., 2008) by reducing the expression of the osteoclastic enzyme cathepsin K. Thus, statins play an important role in both osteoblast activation and osteoclast inhibition, which leads to increased bone mass.

While successive administration of simvastatin has been shown to enhance new bone formation around implants and increase the BIC (Ma et al., 2008; Du et al., 2009; Ayukawa et al., 2010), there is no study, to the best of our knowledge, investigating the impact of short‐term simvastatin administration and subsequent discontinuation on peri‐implant bone. Simvastatin was administered for seven days in the present study, which is shorter than the typical 4 weeks of administration employed in previous studies (Ma et al., 2008; Du et al., 2009; Ayukawa et al., 2010). The amount of trabecular bone around the implant was found to continuously increase in the experimental group up to seven days after simvastatin discontinuation. This effect was more pronounced at the proximal end (tibial growth plate side) than the distal end of the implant. As the half‐life of simvastatin is 2 h, this prolonged effect may be due to residual proteins, such as BMPs, induced by the administration of simvastatin.

On the other hand, following discontinuation of simvastatin on day 7, the amount of trabecular bone around the implant had gradually decreased in the experimental group, which was lower than that in the control group. It is worth noting that the lowest new BV was observed on day 14 after discontinuation of simvastatin and was significantly lower than that in the control group. By day 21, new BV returned to levels comparable with that of the control group, which may reflect a rebound of withdrawal and recovery (Daskalopoulou, 2009).

The underlying mechanism of this reduction is not clear. However, a recent study demonstrated that abrupt termination of statin therapy resulted in a rebound in inflammatory marker levels, such as a rapid increase in interleukin‐6 (IL‐6) levels, in patients with hypercholesterolemia (Li et al., 2006). In this study, simvastatin administration was stopped seven days after implant installation, which coincides with the inflammatory phase following dental implant installation. Therefore, it can be speculated that IL‐6 production was enhanced by simvastatin discontinuation, as IL‐6 is a proinflammatory and bone‐resorbing cytokine (Hapidin et al., 2007) that induces RANKL expression in bone tissues and enhances osteoclastogenesis (Inada and Miyaura, 2010), resulting in the loss of trabecular bone and a decrease in new BV. This rebound phenomenon may be specific to short‐term simvastatin administration, as it was not observed when simvastatin was administered for 4 weeks before discontinuation (Fig. S1). Further studies on the expression of cytokines around the implant are needed to prove this hypothesis.

Results from three‐dimensional morphometrical analyses revealed a regional difference in the effect of statin administration and discontinuation. In particular, it appears that the effect was limited to cancellous bone, as it was not evident in the cortical bone area. One limitation of the current study is that only morphological and morphometrical analyses were performed. As the cortical bone area is critical for implant support, the present findings may not directly apply to the actual stability of dental implants. Although the physical strength of dental implants is out of the scope of this study, functional analyses, such as removal torque, could confirm the actual effect of simvastatin on dental implants.

Early bone formation around implants is particularly important for immediate loading cases, and systemic or topical administration of medications that induce osteogenesis, such as simvastatin, may prove to be beneficial. However, long‐term administration of simvastatin for the purpose of dental implants is not ideal in most countries. Moreover, short‐term administration of simvastatin (followed by its discontinuation) could be harmful, as it may affect the initial stability of dental implants at the critical phase. Despite these shortcomings, use of simvastatin for dental implant therapies may be justified in some cases (e.g., in patients with lower bone mineral density or other compromising conditions).

In conclusion, even short‐term administration of simvastatin was found to markedly enhance the amount of new bone formed around the implant, suggesting that it may contribute to initial implant stability. However, discontinuation of simvastatin may induce a rebound phenomenon and cause immediate bone loss, especially at the cancellous bone region, suggesting the need for relatively long‐term simvastatin administration for dental implant therapy. Use of simvastatin for dental implant therapy should be determined based on its benefits, as well as its shortcomings.

## Author's Roles

Study design: XL, JY, and HK. Study conduct: XL, FW, JY, and YZ. Data analysis: XL, JY, and HK. Data interpretation: XL, FW, AS, and HK. Manuscript preparation: XL and HK.

## Conflict of interest

The authors state that there is no conflict of interest regarding this study.

## Supporting information


**Supplemental Figure S1.** Change in new bone volume in the long‐term administration (continuously for 4 weeks) group. Animals were analyzed at 7, 14, 21, 28, 35, and 42 days after implantation. Simvastatin was discontinued on day 28 after implantation. Regardless of whether simvastatin was discontinued, new bone volumes had increased more in the experimental group, compared with the control group.
