# Supplementary figures and images for: Discontinuation of simvastatin leads to a rebound phenomenon and results in immediate peri‐implant bone loss
Source: Clin Exp Dent Res. 2016 Mar 18;2(1):65–72. doi: 10.1002/cre2.23 (PMC5839186; doi:10.1002/cre2.23)

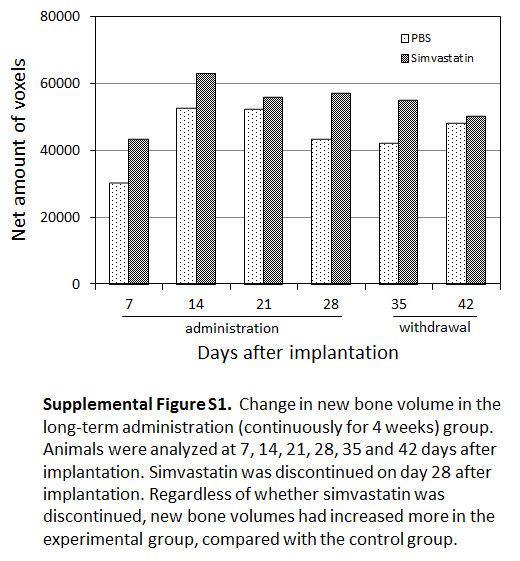

Supplement: Supplementary file 1 — Supplemental Figure S1. Change in new bone volume in the long‐term administration (continuously for 4 weeks) group. Animals were analyzed at 7, 14, 21, 28, 35, and 42 days after implantation. Simvastatin was discontinued on day 28 after implantation. Regardless of whether simvastatin was discontinued, new bone volumes had increased more in the experimental group, compared with the control group. [file CRE2-2-65-s001.tif]
